# Compound muscle action potential (CMAP) scan examination of paretic and contralateral muscles reveals motor unit alterations after stroke

**DOI:** 10.1007/s11427-022-2308-8

**Published:** 2023-05-29

**Authors:** Maoqi Chen, Zhiyuan Lu, Xiaoyan Li, Ya Zong, Qing Xie, Sheng Li, Ping Zhou

**Affiliations:** 1 School of Rehabilitation Science and Engineering, University of Health and Rehabilitation Sciences, Qingdao 266071, China; 2 Department of Neurology, Medical College of Wisconsin, Milwaukee WI 53226, USA; 3 Fischell Department of Bioengineering, University of Maryland at College Park, College Park MD 20742, USA; 4 Department of Rehabilitation Medicine, Ruijin Hospital, Shanghai Jiao Tong University School of Medicine, Shanghai 200025, China; 5 Department of Physical Medicine and Rehabilitation, University of Texas Health Science Center, Houston TX 77030, USA; 6 TIRR Memorial Hermann Research Center, TIRR Memorial Hermann Hospital, Houston TX 77030, USA

**Keywords:** compound muscle action potential (CMAP) scan, stroke, motor unit, muscle weakness, step index (STEPIX), first dorsal interosseous (FDI)

## Abstract

This study presents a novel compound muscle action potential (CMAP) examination of motor unit changes in paretic muscle post stroke. CMAP scan of the first dorsal interosseous (FDI) muscle was performed bilaterally in 16 chronic stroke subjects. Various parameters were derived from the CMAP scan to examine paretic muscle changes, including CMAP amplitude, D50, step index (STEPIX) and amplitude index (AMPIX). A significant decrease in CMAP amplitude and STEPIX was observed in paretic muscles compared with contralateral muscles (CMAP amplitude: paretic (9.0±0.5) mV, contralateral (11.3±0.9) mV, *P*=0.024; STEPIX: paretic 101.2±7.6, contralateral 121.9±6.5, *P*=0.020). No significant difference in D50 and AMPIX was observed between the paretic and contralateral sides (*P*>0.05). The findings revealed complex paretic muscle changes including motor unit degeneration, muscle fiber denervation, reinnervation and atrophy, providing useful insights to help understand neuromuscular mechanisms associated with weakness and other functional deterioration post stroke. The CMAP scan experimental protocols and the applied processing methods are noninvasive, convenient, and automated, offering practical benefits for clinical application.

## INTRODUCTION

As one of leading causes of adult disability and death, stroke has a dramatic negative influence on health-related quality of life. Stroke survivors suffer from various physical symptoms on the affected side (contralesional to brain injury) of the body including spastic hypertonia, weakness, and impaired movement coordination. Weakness or muscle strength deficits post stroke has been identified to be a major factor leading to motor function impairment or disability (Bourbonnais and Noven, 1989). For example, gait performance was reported to be significantly affected by lower limb muscle strength deficits (Bohannon, 1991). Hand grip strength was found to be a good predictor or indicator of arm function recovery for stroke survivors (Boissy et al., 1999; Harris and Eng, 2007).

Motor unit is the basic structure-function element of the neuromuscular system and the final pathway for muscle force generation and control. A range of electromyography (EMG) studies have been performed to examine motor unit alterations contributing to muscle weakness after stroke, including single fiber EMG (Chang, 1998; Lukács et al., 2009; Yao et al., 2018), needle EMG (Brown and Snow, 1990; Dattola et al., 1993; Lukács, 2005; Yao et al., 2018), fine wire EMG (Gemperline et al., 1995; Mottram et al., 2014), macro-EMG (Lukács et al., 2008), conventional surface EMG (Bhadane et al., 2016; Li et al., 2014a; Suresh et al., 2015; Zhang et al., 2017a; Zhang et al., 2017b; Zhou et al., 2013) and electrode array surface EMG (Conrad et al., 2017; Hu et al., 2016; Kallenberg and Hermens, 2009; Li et al., 2013; Li et al., 2015; Liu et al., 2022; Yao et al., 2015). A collective of findings has been reported revealing complex structural and control property changes after stroke. In addition to different types of voluntary EMG analysis, motor unit number estimation (MUNE) has been applied to examine paretic muscles in stroke survivors by stimulating motor nerves and recording compound muscle action potentials (CMAPs) (Arasaki et al., 2006; Choi et al., 2007; Hara et al., 2000; Hara et al., 2004; Kouzi et al., 2014; Liu et al., 2023; McComas et al., 1973). While varying degrees of motor unit loss have been reported in stroke by different MUNE methods (such as multipoint stimulation MUNE (Arasaki et al., 2006) and F wave MUNE (Li et al., 2016a)), the methodology limitations of the MUNE should be acknowledged. Specifically, MUNE is routinely calculated as the ratio of the CMAP to the mean motor unit action potential (MUAP) measure, estimated from a motor unit sample. Therefore, the performance of MUNE can be compromised by a biased sample of motor units for mean MUAP measure, and alterations as well that occurs due to probabilistic and overlapping axon thresholds (DeForest et al., 2018). In addition, most previous MUNE methods are laborious and time consuming, not suitable for clinical application.

The objective of this study was to investigate motor unit degeneration and remodeling changes post stroke using a novel CMAP scan analysis. The CMAP scan is a detailed curve between stimulating current intensity and CMAP amplitude established from a large number of stimuli to the motor nerve (Blok et al., 2007). Since the stimuli can cover from subthreshold to supramaximal intensities thus gradually activating/deactivating all motor units, the CMAP scan can be most representative of the muscle’s motor unit pool. In this study, CMAP scans were recorded from the first dorsal interosseous (FDI) muscles of paretic and contralateral hands. Convenient but physiologically meaningful CMAP scan parameters were derived and compared between the two sides. To the best of our knowledge, this is the first study of applying the CMAP scan technique in stroke population. The findings revealed motor unit loss and muscle fiber denervation/reinnervation/atrophy in paretic muscles, providing useful insights to help understand underlying mechanisms of stroke induced functional deterioration and impairment in paretic muscles. This in turn promises to guide appropriate rehabilitation strategies for improving motor function after stroke.

## RESULTS

### CMAP amplitude and intensity of stimulating current

**Figure 1:**
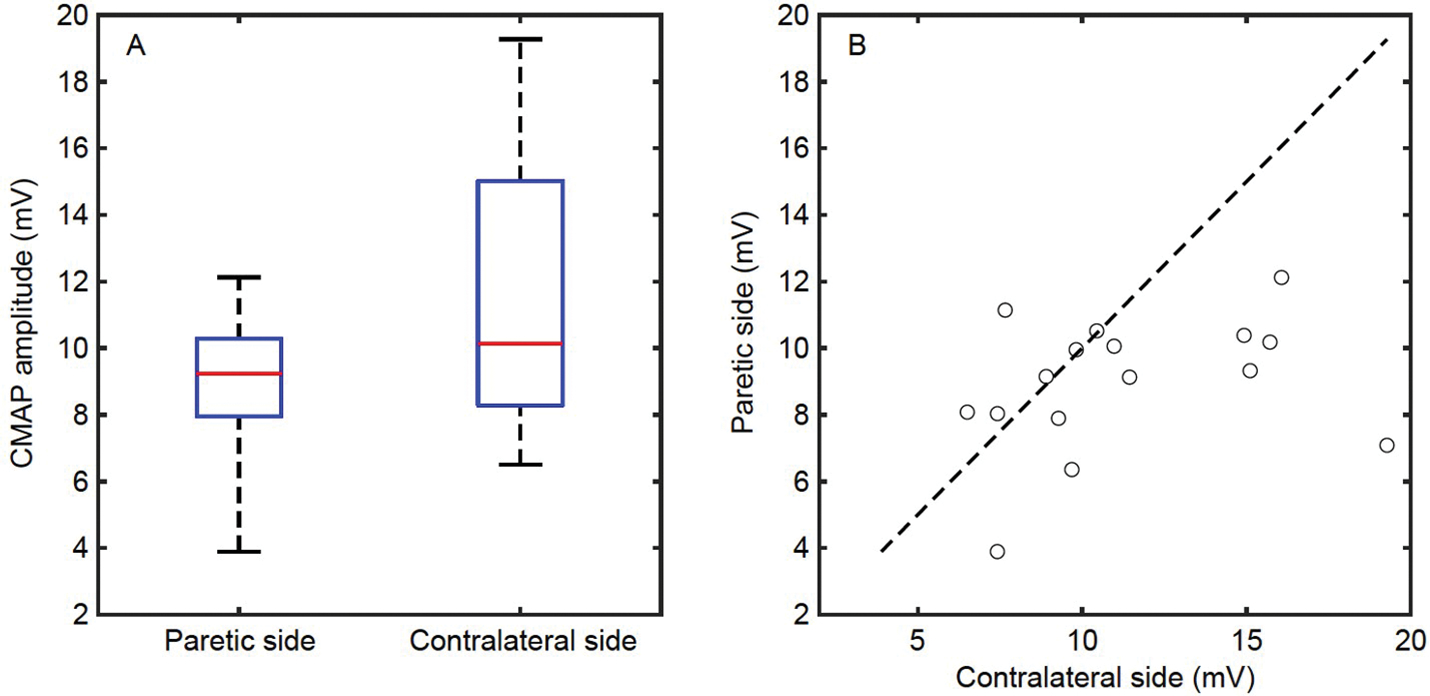
Maximum CMAP amplitude of the FDI muscle. A, The maximum, minimum, and quartiles of maximum CMAP amplitude of the two sides. B, Maximum CMAP amplitude of each subject. Each circle refers to one subject. Subjects with smaller maximum CMAP amplitude on their paretic side locate below the dashed line, and *vice versa*.

All the recruited subjects completed the CMAP scan experiments. Pain and discomfort associated with applied electrical stimulations were reported as minor or tolerable. The maximum CMAP amplitude of the paretic side was (9.0±0.5) mV (in the format of mean±standard error), which was significantly smaller (*P*=0.024) than that of the contralateral side ((11.3±0.9) mV), as shown in Figure 1A. The corresponding intensity of the stimulating currents (denoted as S100) was (19.9±1.5) mA for the paretic side and (19.4±1.7) mA for the contralateral side (*P*=0.877). However, the minimum intensity of the stimulating current required to evoke a visible motor unit activity (denoted as S0) was significantly smaller on the contralateral side ((10.1±1.0) mA for the paretic side and (8.6±1.0) mA for the contralateral side, *P*=0.023).

As shown in Figure 1B, 10 out of 16 subjects had a smaller maximum CMAP amplitude on their paretic side. No significant correlation was observed between the two sides (*P*=0.263). The ratio of individual subject’s maximum CMAP amplitude of the paretic side to the contralateral side was 0.853±0.070, which was not correlated with the functional assessments including the hand function score of Fugl-Meyer assessment (Fugl-Meyer et al., 1975), the hand function of Chedoke-McMaster stroke assessment (Gowland et al., 1993), the ratio of grip and pinch force of the paretic side to the contralateral side (*P*=0.301).

### D50 of CMAP scan curve

**Figure 2:**
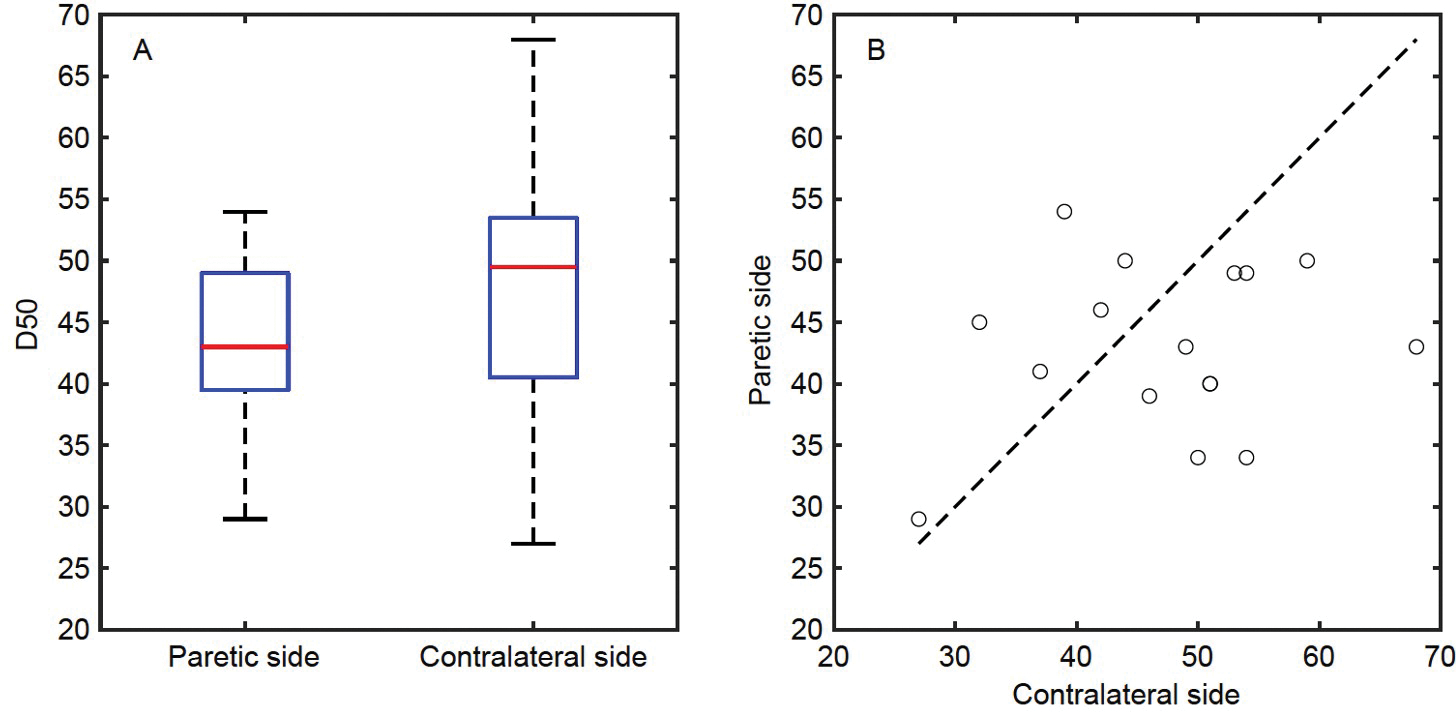
D50 of the FDI muscle. A, The maximum, minimum, and quartiles of D50 of the two sides. B, D50 of each subject. Each circle refers to one subject. Subjects with smaller D50 on their paretic side locate below the dashed line, and *vice versa*.

D50 is an index to quantify CMAP scan discontinuity (Sleutjes et al., 2014). Figure 2B shows the D50 values of the FDI muscle on each subject’s paretic and contralateral sides, respectively. The value of D50 was 42.9±1.7 for the paretic side and 47.2±2.6 for the contralateral side (Figure 2A). No significant difference (*P*=0.140) or correlation (*P*=0.485) was observed between the two sides. The ratio of individual subject’s D50 of the paretic side to the contralateral side was 0.945±0.060, which was not correlated with the functional assessments (*P*=0.180).

### STEPIX and AMPIX of CMAP scan curve

**Figure 3:**
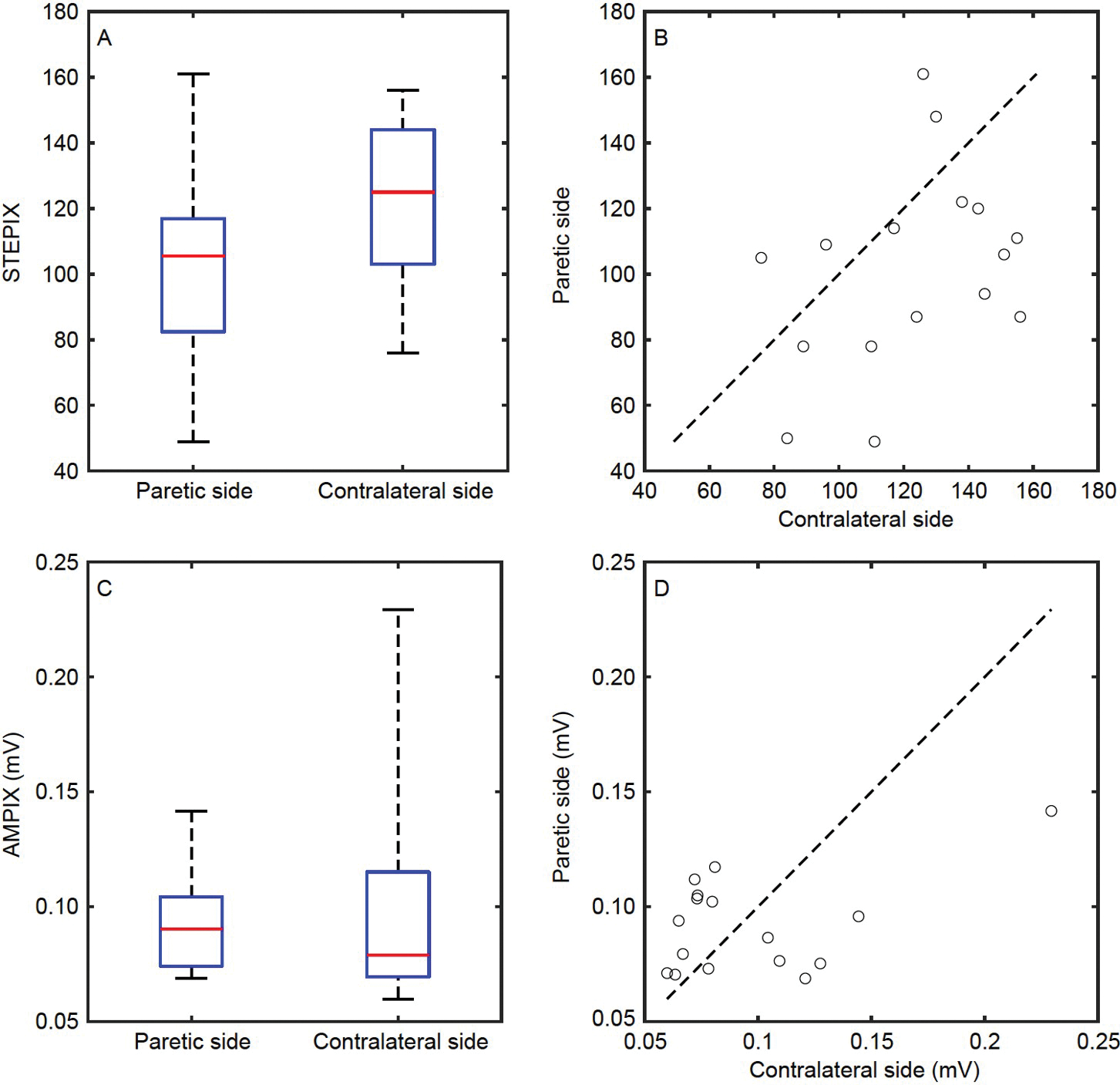
STEPIX and AMPIX of the FDI muscle. A, The maximum, minimum, and quartiles of STEPIX of the two sides. B, STEPIX of each subject. C, The maximum, minimum, and quartiles of AMPIX of the two sides. D, AMPIX of each subject. Each circle in (B) and (D) refers to one subject. Subjects with smaller STEPIX or AMPIX on their paretic side locate below the dashed line, and *vice versa*.

Step index (STEPIX) is an index for the number of motor units, and amplitude index (AMPIX) is an index for motor unit size (Nandedkar et al., 2022). The value of STEPIX was 101.2±7.6 for the paretic side and 121.9±6.5 for the contralateral side (Figure 3A). As shown in Figure 3B, 12 out of 16 subjects had a smaller STEPIX on their paretic side. Statistically, STEPIX of the paretic side was smaller than that of the contralateral side (*P*=0.020). No significant correlation was observed between the two sides (*P*=0.169). The ratio of individual subject’s STEPIX of the paretic side to the contralateral side was 0.849±0.067, which was not correlated with the functional assessments (*P*=0.559).

AMPIX was (0.092±0.005) mV for the paretic side and (0.097±0.011) mV for the contralateral side (Figure 3C and D). No significant difference (*P*=0.634) or correlation (*P*=0.088) was observed between the two sides. The ratio of individual subject’s AMPIX of the paretic side to the contralateral side was 1.060±0.089, which was not correlated with the functional assessments (*P*=0.169).

**Figure 4:**
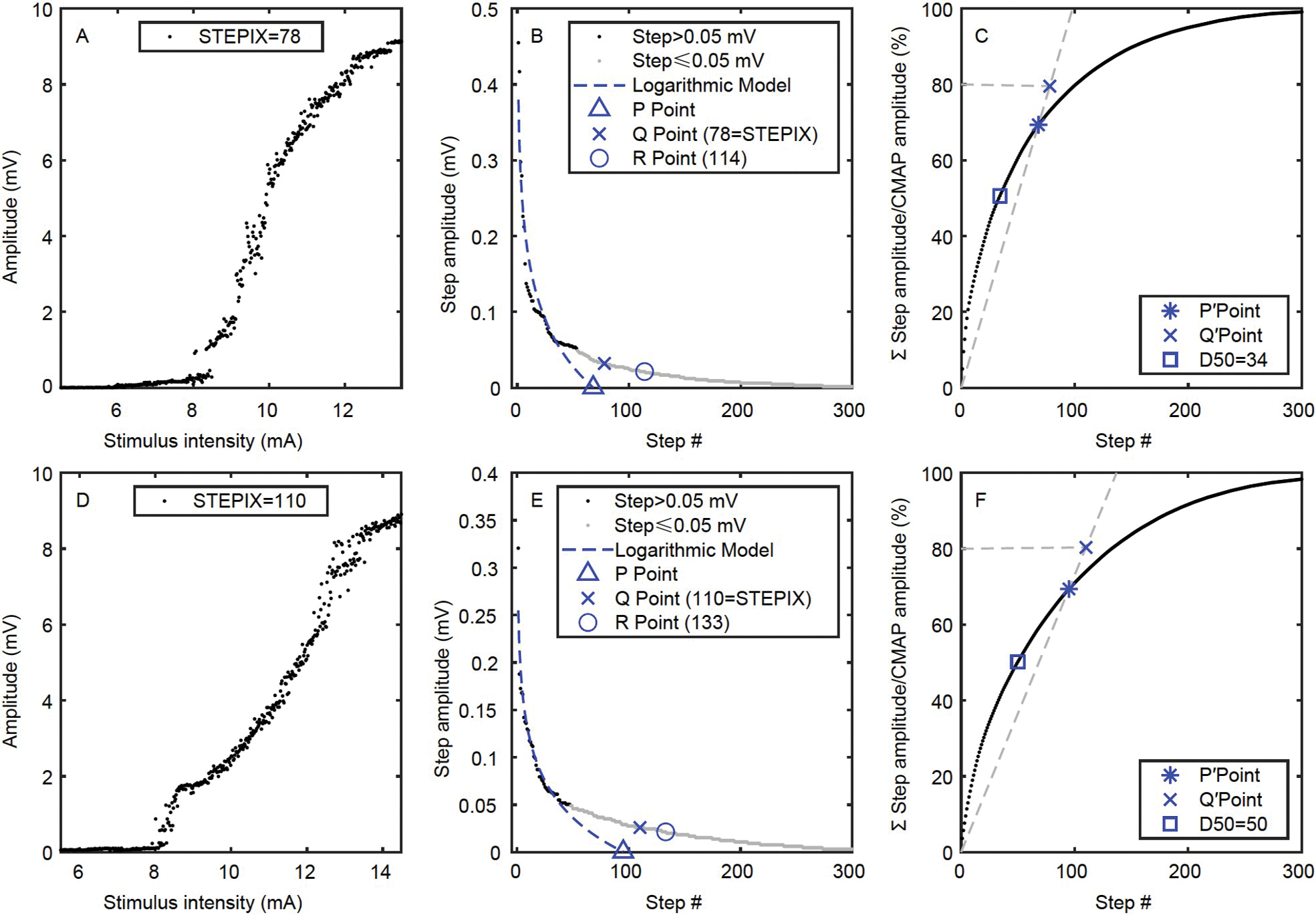
The CMAP scan curve, STEPIX, and D50 of a subject whose paretic side had a smaller STEPIX. A, The CMAP scan curve of the paretic side. B, The curve of sorted steps from the paretic side. C, The curve of accumulative sum of steps from the paretic side. D, The CMAP scan curve of the contralateral side. E, The curve of sorted steps from the contralateral side. F, The curve of accumulative sum of steps from the contralateral side.

**Figure 5:**
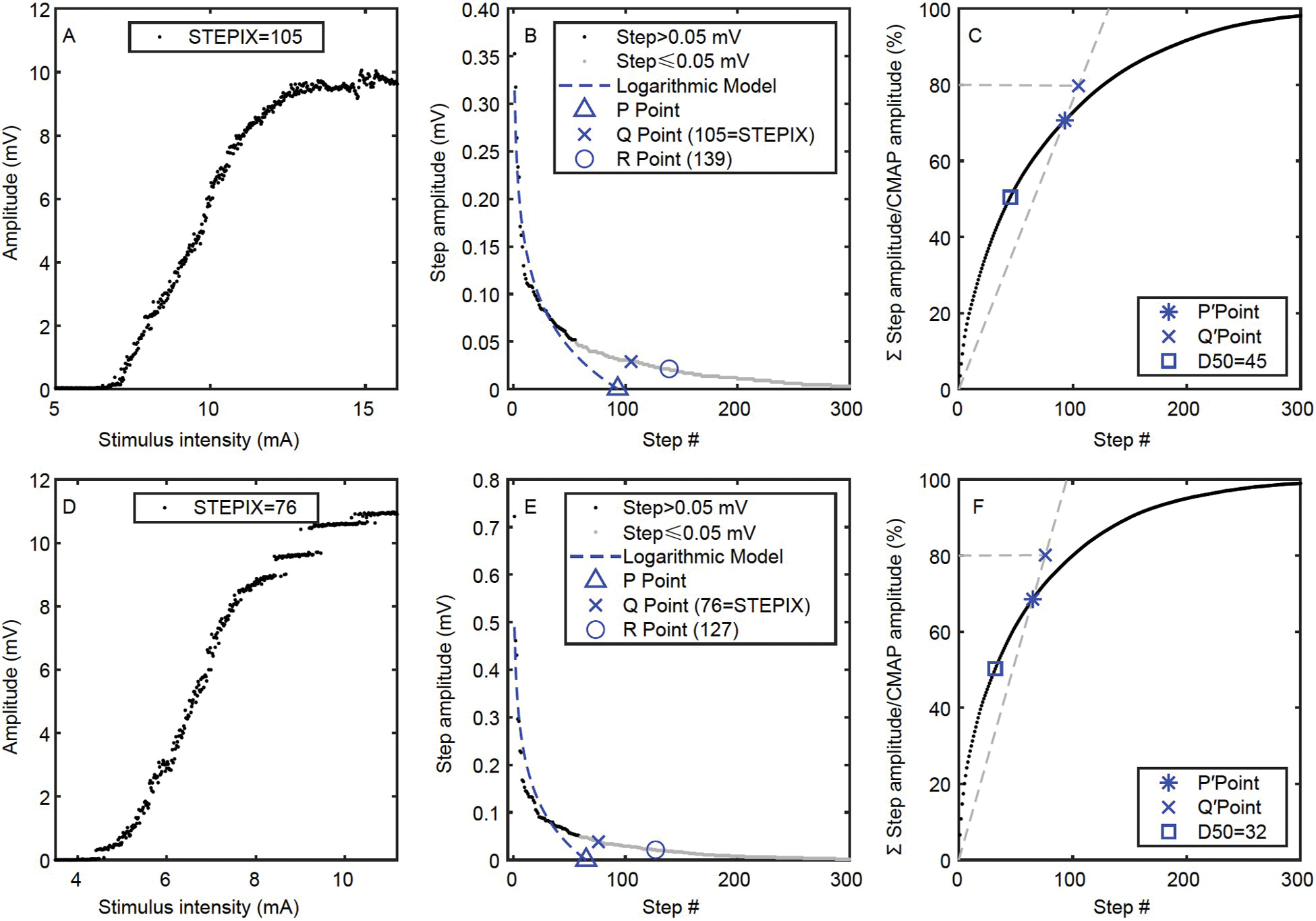
The CMAP scan curve, STEPIX, and D50 of a subject whose contralateral side had a smaller STEPIX. A, The CMAP scan curve of the paretic side. B, The curve of sorted steps from the paretic side. C, The curve of accumulative sum of steps from the paretic side. D, The CMAP scan curve of the contralateral side. E, The curve of sorted steps from the contralateral side. F, The curve of accumulative sum of steps from the contralateral side.

Figure 4 shows the CMAP scan data of a subject whose paretic side had a smaller STEPIX while similar maximum CMAP amplitude was observed compared with the contralateral side. Figure 5 shows another subject whose paretic side had a smaller maximum CMAP amplitude but larger STEPIX compared with the contralateral side.

## DISCUSSION

### Two approaches in CMAP scan processing

This study presents a novel CMAP examination of paretic muscles after stroke. A primary advantage of CMAP scan is its capacity in providing a comprehensive assessment of motor unit population in a muscle. As discussed in (Zhou, 2022), there are different approaches to CMAP scan processing. One approach is direct estimation of the motor unit number from the CMAP scan. This has resulted in MUNE methods which are different from routine calculation of the ratio of CMAP to mean MUAP. Typical development includes Bayesian MUNE (Ridall et al., 2006) and MScanFit MUNE (Bostock, 2016; Jacobsen et al., 2018). The other approach of CMAP scan processing is to characterize motor unit number and size changes through indirect or index parameters. Typical examples include D50 (Sleutjes et al., 2014), STEPIX and AMPIX (Nandedkar et al., 2022), and CMAP distribution index (Lu et al., 2023). Although these parameters do not provide absolute motor unit number, they can be sensitive to changes in motor unit number. As the primary goal of MUNE is usually to compare or track motor unit number changes in either cross-sectional or longitudinal studies, these indirect parameters derived from CMAP scan also serve as an appropriate alternative for this purpose. The usefulness of indirect parameters has also been demonstrated in the examination of paretic muscle changes after stroke, as performed in this study. The calculation of the indirect parameters is straightforward and quick to implement, an attractive feature for clinical application.

### Motor unit loss after stroke

The findings of the current CMAP scan study add new evidence of motor unit changes due to stroke, manifested as significantly smaller CMAP amplitude and STEPIX in the paretic muscle compared with the contralateral muscle. This is in line with previous MUNE studies that reported varying degrees of CMAP and motor unit number reduction after stroke (Arasaki et al., 2006; Choi et al., 2007; Hara et al., 2000; Hara et al., 2004; Kouzi et al., 2014; Li et al., 2016a; Liu et al., 2023; McComas et al., 1973). The minimum current intensity (S0) for evoking a motor unit was significantly higher for the paretic muscle compared with the contralateral side, suggesting altered axon excitability properties after stroke (Klein et al., 2020; Turan and Zİnnuroğlu, 2020). It is noted that another CMAP scan parameter, D50, was not significantly different between the paretic and contralateral sides. D50 was derived from sorted step amplitudes to quantify discontinuities in the CMAP scan due to motor unit degeneration and muscle fiber reinnervation. D50 has been demonstrated useful in monitoring neuromuscular diseases (such as amyotrophic lateral sclerosis, ALS) with significant motor unit loss (Sleutjes et al., 2020; Sleutjes et al., 2021). However, it has been reported less sensitive than MScanFit in assessing motor unit loss (Sirin et al., 2019; Zong et al., 2021). In the current study, STEPIX also appeared to be more sensitive than D50 in detecting paretic muscle changes. The significant reduction in STEPIX but not in D50 may imply a mild to moderate level of motor unit loss in the tested stroke subjects. It should be acknowledged that although electrophysiological studies in stroke survivors provide evidence of motor unit loss suggesting transsynaptic spinal motor neuron degeneration after a brain lesion, supportive data is lacking in post-mortem histological studies. Contrary to reduction of the number of motor units on the hemiparetic side in stroke patients, no significant difference was observed in anterior horn cell numbers between the affected and unaffected sides in stroke, while the trans-sectional areas were significantly smaller associated with the hemispheric lesion side (Qiu et al., 1991; Terao et al., 1997). Therefore, it still remains to be determined how different motor unit components might be affected following a cerebral lesion, a somewhat controversial question due to conflicting evidence from electrophysiological and histological studies.

### The effects of muscle fiber atrophy

In addition to STEPIX of the CMAP scan, motor unit number index (MUNIX) (Nandedkar et al., 2004), derived from maximum CMAP and surface EMG at different levels of voluntary contraction, was also used for examination of paretic muscles of stroke (Li et al., 2011; Li et al., 2014b). In fact, according to a recent review (Wright et al., 2023), MUNIX has become the most frequently used method in literature among all kinds of MUNE methods, primarily due to its convenience and least discomfort to tested subjects. When applying MUNIX, the effect of muscle fiber atrophy (MUAP amplitude) should be considered for result interpretation, since the MUNIX reduction could be attributed to not only motor unit loss, but also muscle fiber atrophy (Gomes de Sousa et al., 2019; Li et al., 2012). To overcome the effect of muscle atrophy on MUNIX, a modified MUNIX was proposed which uses a variable proportional to the maximum CMAP area rather than a fixed 20 mV ms for calculation of MUNIX (Li et al., 2016b). However, it remains a dilemma to apply MUNIX or modified MUNIX for examination of stroke patients (Zhou et al., 2016). Compared with MUNIX that requires voluntary muscle contraction, STEPIX can be used for patients who lack voluntary control of their musculature. However, similar to MUNIX, the effect of muscle fiber atrophy on STEPIX should be considered for result interpretation. In this study, significantly lower STEPIX was observed in the paretic muscle compared with the contralateral muscle, but there was no significant difference in AMPIX between the two sides. It is likely that decreased STEPIX after stroke was due to both motor unit loss and muscle fiber atrophy. On one hand, motor unit size could be enlarged due to muscle fiber reinnervation following motor unit degeneration. On the other hand, MUAP amplitude might be decreased by muscle fiber atrophy. These two opposite effects justified the lack of significant difference in AMPIX between paretic and contralateral muscles.

### Functional assessment

Although CMAP scan examination indicated a significant STEPIX reduction in paretic muscles post stroke, we did not find STEPIX changes were correlated with any clinical assessment, such as the grip force and Fugl-Meyer upper limb assessment. This might be due to several factors. First, this study only examined a single FDI muscle, while clinical assessment of arm or hand function usually involves coordination of different muscles, which is not considered in the current study. For example, in addition to distal hand muscles, forearm muscles also contribute to maximum grip strength, which was not examined in this study. Second, complex neural and muscular changes may occur post stroke including motor unit loss, muscle fiber denervation/reinnervation, and motor unit control property alteration, collectively affecting motor function. Both central and peripheral factors can be altered after stroke contributing to muscle weakness, which can compromise a single factor’s correlation with clinical assessment. Despite non-significant correlation with routine functional assessments, examination of STEPIX and AMPIX still has clinical relevance in that it can help understand motor unit alterations contributing to weakness of the examined muscle. This will help develop appropriate treatment strategies targeting specific motor unit properties. For example, if weakness proves to arise primarily from motor unit loss, treatment strategies can be focused on enhancing the natural reinnervation process through exercise and electrical stimulation to maintain muscle strength.

### Summary, limitations and future work

In summary, this study presents a novel CMAP scan examination of paretic muscles in 16 stroke subjects. There was a significant reduction in CMAP amplitude and STEPIX in the paretic side compared with the contralateral side, suggesting motor unit loss and muscle fiber denervation/reinnervation/atrophy changes after stroke. An attractive feature of the performed analyses is their fast and convenient character, offering practical benefits in clinical environment. A limitation of the study is that the correlation between the CMAP scan parameters and the single muscle weakness was not examined since the maximum voluntary contraction force of the FDI muscle was not recorded using a standardized load cell setup. In addition, there is a lack of comparison between the CMAP scan and other notable existing methods (such as single fiber EMG, MUAP analysis, high density surface EMG). At this stage, the relative contribution of different motor unit property changes to muscle weakness is still uncertain. It is likely that this relative contribution varies in different stroke patients. Our future work will involve in differentiation of changes in specific motor unit components and quantification of their contributions to muscle weakness after stroke. This requires a combination of CMAP scan and other electrophysiological techniques, which can supplement each other for a comprehensive examination of paretic muscles after stroke.

## MATERIALS AND METHODS

### Subjects

Sixteen subjects (10 males, 6 females) with post-stroke hemiplegia (8 subjects on the left and 8 subjects on the right) were recruited in this study. Grip force of each hand was measured using Jamar Plus+ digital hand dynamometer (Patterson Medical, USA), and pinch force was measured using PG-60 pinch gauges (B&L Engineering, USA). The Fugl-Meyer upper limb assessment (FM) and Chedoke-McMaster stroke assessment (CM) were performed on each subject by an experienced therapist. Our subjects covered a wide range from mild to severe impairment, with their FM score ranging from 10 to 58 (a full score of 66), and the score of the hand part of FM ranged from 0 to 14 (a full score of 14). Our subjects also covered the full range of CM hand function (i.e., from stage 1 to stage 7). Each subject had only one stroke more than 6 months before the experiment, and the conduction velocity of the ulnar nerve was in the normal range for both sides. Details of subjects can refer to Table 1.

### CMAP scan experiment

**Figure 6:**
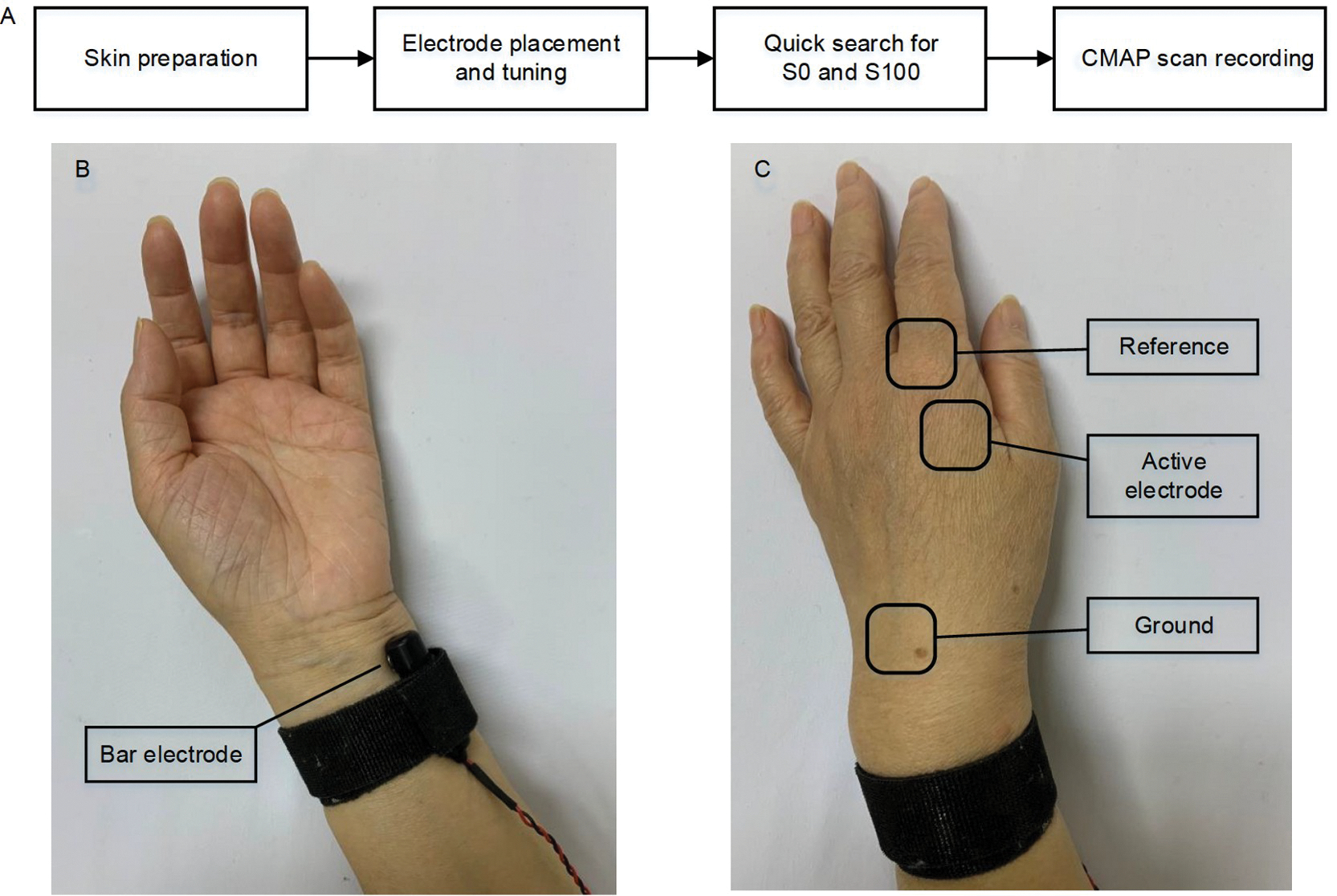
Experimental protocols of CMAP scan of the FDI muscle. A, Procedures of a CMAP scan experiment. B, Electrode placement for current stimuli. C, Electrode placement for EMG recording. It also shows the hand posture in the experiment.

CMAP scan was performed on both hands of the subject in a random order. In the experiment, the subject was seated comfortably in a chair or wheelchair with his/her testing hand relaxed on a table in the pronation position. An EMG system (UltraPro S100, Natus Neurology Incorporated, USA) was employed to generate current stimuli and record the CMAP or M wave response of the FDI muscle. Figure 6A shows a brief flow chart of CMAP scan recording. Stimulating currents were delivered to the ulnar nerve by using a bar electrode placed proximal to the wrist with its two contact surfaces (9 mm in diameter and 20 mm apart) parallel to the ulnar nerve (Figure 6B). CMAP response was captured by Ag/AgCl electrodes (10 mm in diameter). The active electrode was placed on the FDI muscle belly. The reference was placed on the metacarpophalangeal joint of the index finger. The ground was placed on the dorsal side of the hand (Figure 6C). Both recording and stimulating electrodes were carefully tuned in order to optimize electrode positions where the largest CMAP amplitude can be evoked with a relatively low stimulating current intensity. A CMAP was obtained by continuously increasing the current intensity until the maximum motor response was reached. This was confirmed by no enlargement in the peak-to-peak CMAP amplitude with further increased stimulating current intensities.

A total of 1,000 stimuli were delivered in each CMAP scan recording. Each stimulus had a constant intensity, lasted for 0.2 ms, and was delivered every 500 ms. The current intensity of each stimulus decreased linearly from the first stimulus to the last one. Specifically, the stimulus with the highest current intensity was delivered at the beginning, activating all motor units of the FDI muscle. The stimulus with the lowest current intensity was delivered at last, not able to activate any of the motor units. The values of the maximum and minimum current intensities were determined for each examined muscle prior to the CMAP scan recording. CMAP response of each stimulus was recorded and then down-sampled to 500, generating a CMAP scan curve containing 500 data points.

### Calculation of D50, STEPIX, and AMPIX

STEPIX and AMPIX were calculated using a customized Matlab script based on their definitions and recommended settings (Nandedkar et al., 2022). A brief introduction of our algorithms is provided below. Firstly, the amplitude difference between every two adjacent data points on the sorted CMAP scan curve was calculated and defined as steps. Secondly, the R point was located on the curve of sorted steps (the black and grey data points in Figure 4B) by looking for the data point with the smallest amplitude that is larger than 0.02 mV. Thirdly, a logarithmic model (the dashed line in Figure 4B) was built up using steps with an amplitude greater than 0.05 mV (the black data points in Figure 4B), and the mean amplitude of the model’s output (MAM) was estimated. Fourthly, the ratio of 80% of the maximum CMAP amplitude to the MAM was taken as the abscissa of the Q point (Figure 4B) and Q′ point (Figure 4C). STEPIX is the smaller abscissa of the Q point and R point. And AMPIX is the ratio of the maximum CMAP amplitude to STEPIX. The minimum number of steps that build up 50% of the maximum CMAP amplitude is defined as D50 (Sleutjes et al., 2014).

### Statistical analysis

Paired *t*-test was applied to compare normally distributed CMAP scan data of the paretic side and the contralateral side, including the maximum CMAP, D50, and STEPIX. Wilcoxon signed rank test was applied if the data were non-normally distributed (such as S100). Multiple regression was applied to analyze the correlation between CMAP scan data and functional assessment data including grip force, pinch force, and hand function scores. All the analysis was performed by using Matlab (R2022). Significance level was 0.05.

## Figures and Tables

**Table 1:** Demography of subjects^a)^

Age	Gender	Paretic side	FM	CM	YAS	GF-P (kg)	GF-C (kg)	PF-P (kg)	PF-C (kg)

61	F	R	23/2	2	7.8	5.2	27.3	2.0	6.0
62	F	L	24/8	3	13.6	9.4	30.6	3.0	6.0
50	F	R	17/0	1	0.8	1.8	25.1	1.0	6.0
70	M	R	21/6	2	7.2	9.7	44.8	5.0	9.0
59	F	L	39/7	6	8.7	4.6	25.7	1.5	4.5
80	M	R	26/6	2	6.3	20.7	26.1	4.5	7.0
52	M	R	20/5	2	1.9	4.0	33.1	3.0	7.0
56	F	L	58/14	7	7.8	21.2	32.2	5.0	8.7
47	M	L	48/8	4	1.7	10.7	34.6	3.9	8.1
72	M	L	39/13	6	10.6	10.1	44.5	6.5	12.0
71	M	R	14/2	2	1.1	0	NA	0	NA
50	F	L	24/4	3	7.3	12.6	44.3	5.1	11.5
55	M	R	21/1	3	4.8	1.8	41.7	5.5	9.0
34	M	R	46/11	6	4.1	15.4	29.9	3.5	10.5
72	M	L	10/0	2	NA	0	28.1	0	9.1
62	M	L	15/1	3	3.6	0	29.0	2.7	8.2

a) FM, score of Fugl-Meyer upper limb assessment, in the format of “total score/hand score”; CM, hand stage of Chedoke-McMaster stroke assessment; YAS, years after stroke; GF-P, grip force of the paretic hand; GF-C, grip force of the contralateral hand; PF-P, pinch force of the paretic hand; PF-C, pinch force of the contralateral hand. NA, not available.
